# A smart nanoplatform for enhanced photo-ferrotherapy of hepatocellular carcinoma

**DOI:** 10.3389/fbioe.2022.1022330

**Published:** 2022-09-20

**Authors:** Longguang Tang, Mingjian Ling, Madiha Zahra Syeda, Rui Sun, Minghui He, Qingchun Mu, Xiulong Zhu, Chunming Huang, Liao Cui

**Affiliations:** ^1^ The People’s Hospital of Gaozhou, Guangdong Medical University, Maoming, China; ^2^ Guangdong Provincial Key Laboratory of Research and Development of Natural Drugs, and School of Pharmacy, Guangdong Medical University, Dongguan, China; ^3^ Southern Medical University Shenzhen Stomatology Hospital (Pingshan), Shenzhen, China; ^4^ Department of Pharmacology and Department of Respiratory and Critical Care Medicine of the Second Affiliated Hospital, Zhejiang University School of Medicine, Key Laboratory of Respiratory Disease of Zhejiang Province, Hangzhou, China; ^5^ School of Pharmaceutical Sciences, Guangdong Provincial Key Laboratory of New Drug Screening, Southern Medical University, Guangzhou, China; ^6^ Key Laboratory of Growth Regulation and Translational Research of Zhejiang Province, School of Life Sciences, Westlake University, Hangzhou, China

**Keywords:** GSH, ferrotherapy, PTT, hepatocellular carcinoma, nanoparticle

## Abstract

Hepatocellular carcinoma (HCC) is the third leading cause of cancer-related deaths worldwide. Emerging therapies, such as ferroptosis mediated cancer therapy and phototherapy, offer new opportunities for HCC treatment. The combination of multiple treatments is often more effective than monotherapy, but many of the current treatments are prone to serious side effects, resulting in a serious decline in patients’ quality of life. Therefore, the combination therapy of tumor *in situ* controllable activation will improve the efficacy and reduce side effects for precise treatment of tumor. Herein, we synthesized a GSH-activatable nanomedicine to synergize photothermal therapy (PTT) and ferrotherapy. We utilized a near-infrared dye SQ890 as both an iron-chelating and a photothermal converter agent, which was encapsulated with a GSH-sensitive polymer (PLGA-SS-mPEG), to attain the biocompatible SQ890@Fe nanoparticles (NPs). In the tumor microenvironment (TME), SQ890@Fe NPs showed a GSH-activated photothermal effect that could increase the Fenton reaction rate. Meanwhile, the depletion of GSH could further increase ferroptosis effect. In turn, the increasing radical generated by ferrotherapy could impair the formation of heat shock proteins (HSPs) which could amplify PTT effects by limiting the self-protection mechanism. Overall, the intelligent nanomedicine SQ890@Fe NPs combines ferrotherapy and PTT to enhance the efficacy and safety of cancer treatment through the mutual promotion of the two treatment mechanisms, providing a new dimension for tumor combination therapy.

## Introduction

Ferroptosis is an iron-dependent programmed cell death that is distinguished from other forms of cell death by excessive lipid peroxidation (LPO) on the membrane (Cao et al., 2016; Tarangelo and Dixon, 2016; Hassannia et al., 2019). The ability to elevate iron-catalyzed ROS levels endows ferrotherapy a promising strategy to bypass the drug resistance issue, which is a major challenge in cancer chemotherapy (Yang et al., 2014; Stockwell et al., 2017). Thus, ferroptosis inducing agents have been a focus of intense research recently. The development of ferroptosis-based nanomedicine for cancer therapy has made tremendous progress in the past decade (Shen et al., 2018; Liang et al., 2019). Nevertheless, the ROS-induced cell damage may be resisted by the intracellular redox balancing mechanisms, limiting the therapeutic efficacy of ferrotherapy (Tang et al., 2019; Tian et al., 2021). One such example is GSH, a high concentration reducing agent in TME, that maintains the intracellular redox balance by eliminating oxidative stress (Zheng and Conrad, 2020). Therefore, increasing the generation of ROS by depleting intracellular GSH is considered an effective method to improve the therapeutic effect (Cheng et al., 2021; Xiong et al., 2021). This led to the research and development of promising combination therapies where ferroptosis was equipped with GSH-depletion strategies in cancer therapy (Ou et al., 2021). Moreover, due to the potential synergy between various treatments, the paradigm shift to combination therapy has gained significant attraction (Chen et al., 2019; He et al., 2021a). Advanced understanding of ROS-related signaling pathways has sparked an interest to combine ferroptosis with other treatments, such as chemotherapy (Ma et al., 2017), immunotherapy (Zhang et al., 2019; Liang et al., 2021), and sonodynamic therapy (Zhu et al., 2019). However, in designs that combination therapies improve overall treatment outcomes, there is often a lack of rationales for synergy between therapeutics.

PTT is a promising approach for the cancer therapy due to its intrinsic advantages of low toxicity, minimal invasiveness, negligible drug resistance, high tumor destruction efficiency with fewer side effects and convenient operation (Liu et al., 2019). Upon NIR (near-infrared) light illumination, photothermal conversion agents (PTAs) convert incident light into hyperthermia to physically damage cancer cells. However, HSPs in cancer cells are over-expressed at higher temperature condition, which could trigger the self-protection mechanism in tumor cells and impair both heat-induced injury and downstream biological responses (Wang et al., 2017; Mader et al., 2020). To this end, significant efforts have been made to combine photothermal therapy with HSPs inhibition to improve the therapeutic outcomes of PTT alone (Luo et al., 2017; He et al., 2020; Jiang et al., 2020; Liu et al., 2020; Chang et al., 2021). Interestingly, research suggests that membrane LPO and intracellular ROS accumulation during ferrotherapy is capable to inhibit the HSPs expression (Esterbauer et al., 1991; Kaur et al., 1997; Gürbüz and Heinonen, 2015), thus preventing the heat-induced self-protection mechanism. By contrast, the Fenton reaction rate, according to the Arrhenius equation, increases about 4-fold when the ambient temperature rises from 20°C to 50°C (Sun et al., 2009; Jiang et al., 2020). Owing to these researches, we hypothesized that combining ferrotherapy and PTT could yield promising results in terms of maximizing the respective therapeutic effect for improved cancer therapy.

Squaraine dyes are a less explored class of organic dyes with strong and narrow absorption bands in the NIR region (Ilina et al., 2020). Leveraging from the merits of advanced strategies in probe design to overcome their nucleophilic-attack susceptibility, and new synthetic modifications, squaraine dyes have attracted much attention in biomedical imaging and therapy (Yao et al., 2020; He et al., 2021b).

**FIGURE 1:**
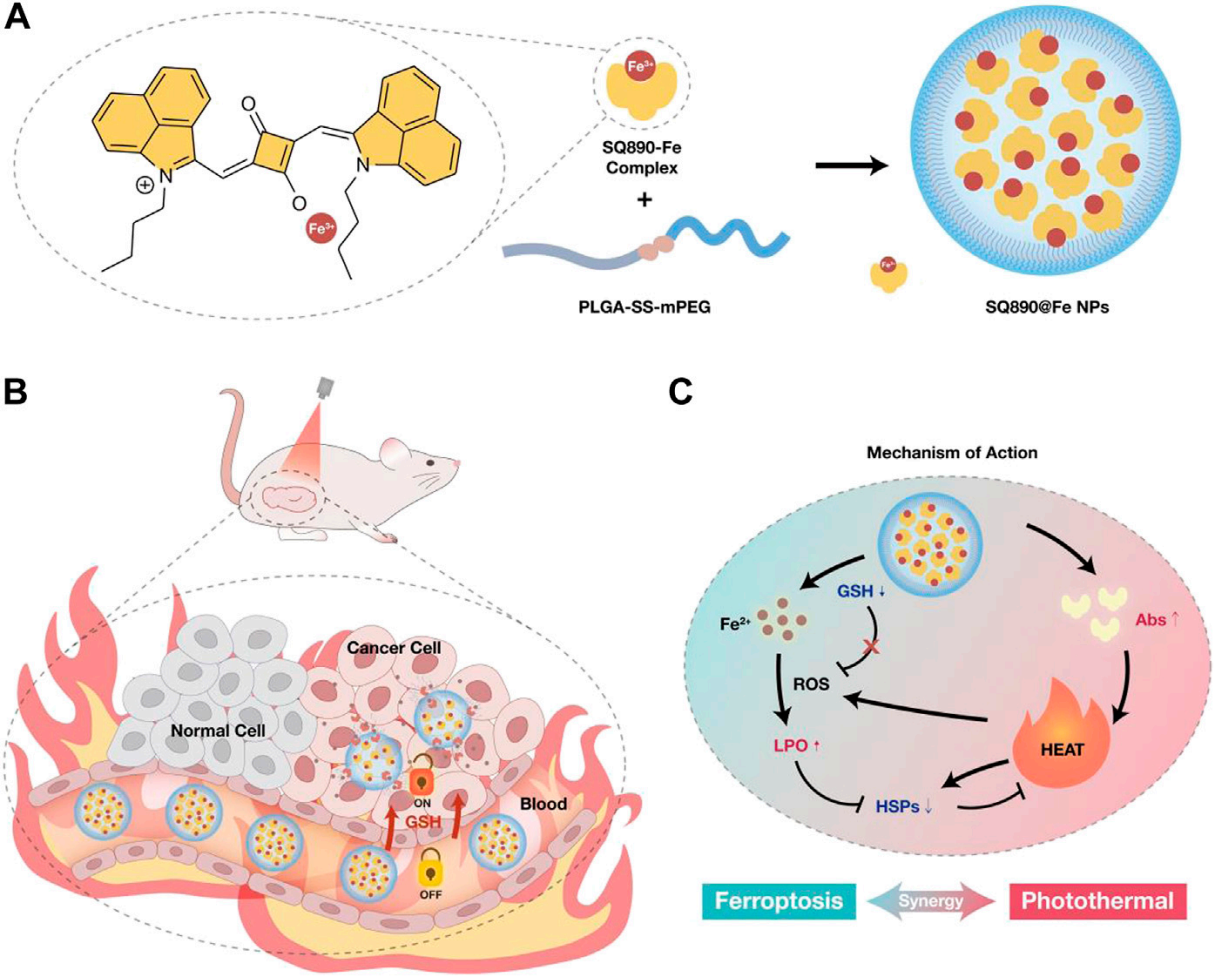
**(A)** Schematic of the preparation of SQ890-Fe^3+^ complexes encapsulated with GSH-sensitive PLGA-SS-mPEG to attain SQ890@Fe NPs nanoparticles. **(B)** The *in vivo* behavior of SQ890@Fe NPs in a subcutaneous patient-derived tumor xenograft model of hepatocellular carcinoma (PDX^HCC^). **(C)** The mechanism of mutually-benefited combination of GSH-activated ferrotherapy and PTT for HCC.

Herein, we designed an iron (III)-coordinated Squaraine dye (SQ890) encapsulated with PLGA-SS-mPEG, for combined GSH activatable ferrotherapy and PTT (Figure 1). The absorption peak of the iron-coordination dyes (SQ890-Fe complexes) dropped significantly, leading to the photothermal effects diminish. However, the absorption peak of SQ890 at 890 nm and its photothermal effects can be recovered by adding GSH. Then, the modified structure (SQ890-Fe^3+^) was encapsulated into nanoparticles (NPs) with PLGA-SS-mPEG, which is a GSH sensitive polymer, for tumor *in situ* release of iron ion. Due to the high concentration of GSH in TME, the Fe^3+^ ions were reduced to Fe^2+^ ions which were dissociated from SQ890-Fe complexes and induced ferroptosis. The absorption peak and photothermal effect of SQ890 were restored at the same time. The photothermal effect and consumption of intracellular GSH could promote the ferrotherapy which in turn improves the therapeutic outcomes of PTT by preventing the generation of HSPs. The mutually beneficial effects of ferrotherapy and PTT were confirmed *in vitro* and *in vivo*, indicating promising prospects for considering combination therapy in the treatment of cancer.

## Results and discussion

### Preparation and characterization of SQ890-Fe^3+^ and SQ890@Fe NPs

**FIGURE 2:**
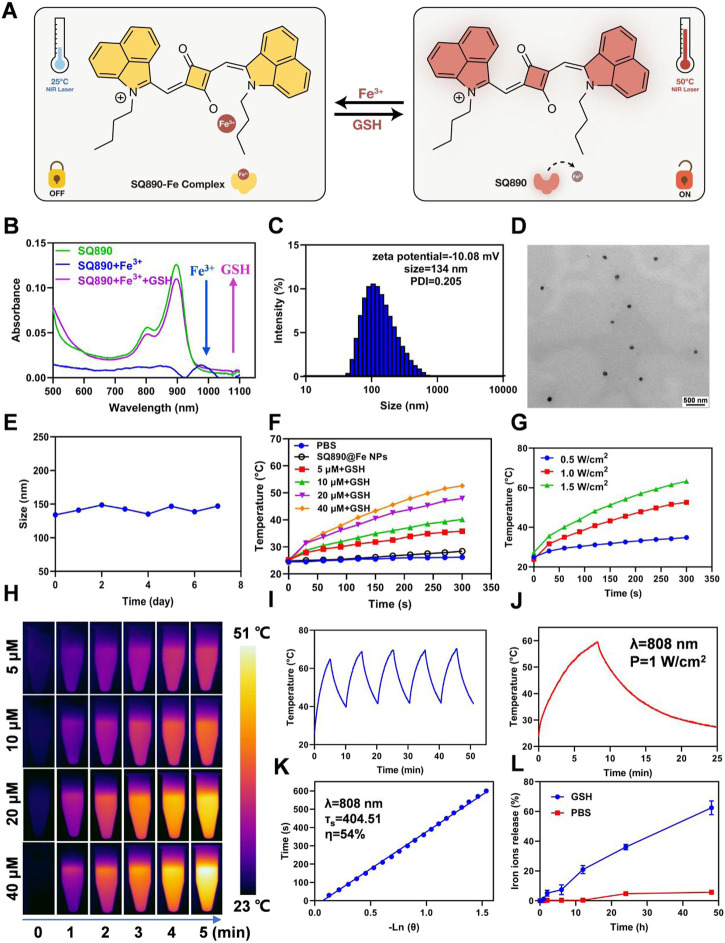
Characterization of SQ890@Fe NPs. **(A)** Under the action of GSH, the photothermal effect of SQ890-Fe was activated, and Fe^3+^ was reduced to Fe^2+^, which was released and lead to the occurrence of Fenton reaction and Ferroptosis. **(B)** The absorption spectra of SQ890 before and after treating with Fe^3+^ ions, and Fe^3+^ ions plus GSH. **(C,D)** Size distribution **(C)** and TEM images **(D)** of SQ890@Fe NPs. Scale bar: 500 nm. **(E)** Stability of SQ890@Fe NPs during 7 days. **(F,G)** Photothermal-heating curves **(F)** and infrared thermal images **(H)** of SQ890@Fe NPs at various concentrations with/without adding GSH (10 mM) under 808 nm laser illumination (1 W cm^−2^) for 5 min. **(H)** The photothermal-heating curves of SQ890@Fe NPs (40 μM) + GSH (10 mM) under different power laser irradiation. **(I)** Photothermic stability of SQ890@Fe NPs + GSH under 808 nm laser illumination for five cycles. **(J,K)** Heating and cooling curves of SQ890@Fe NPs + GSH **(J)** and linear relationship between cooling time and -ln (θ) **(K)**. **(L)** The Fe ions released from SQ890@Fe NPs under the GSH (10 μM) condition.

We first synthesized the SQ890 molecules as described previously, through a three-step reaction, namely electrophilic substitution, Grignard Reaction, and condensation reaction (Ling et al., 2022). Then SQ890-Fe^3+^ was prepared by mixing SQ890 with FeCl_3_ in THF solution. In our hypothesis, under the action of GSH, the photothermal effect of SQ890-Fe will be activated, and Fe^3+^ will be reduced to Fe^2+^, which will be released and lead to the occurrence of Fenton reaction and Ferroptosis (Figure 2A). The ability of SQ890 to chelate Fe ions was evaluated by observing the absorption spectra changes of SQ890. Results showed that the addition of Fe^3+^ ions tremendously reduced the absorbance of SQ890 at 890 nm, indicating the formation of SQ890-Fe^3+^ complexes (Figure 2B), and the addition of Fe^2+^ ions to SQ890 did not change the absorbance (Supplementary Figure S1). Interestingly, after treating with GSH, the Fe^3+^ ions were reduced to Fe^2+^ ions, and the diminished absorption peak of SQ890-Fe^3+^ complexes at 890 nm was recovered. According to the hard and soft acids and bases (HSAB) principle, the SQ890-Fe^3+^ complexes could be formed due to the hard-hard interaction between SQ890 molecules and Fe^3+^ ions. (Ho et al., 1978). To obtain good biocompatibility, SQ890-Fe^3+^ complexes were encapsulated into nanoparticles (SQ890@Fe NPs) through a nanoprecipitation method by the self-assembling of SQ890-Fe^3+^ and the GSH sensitive polymer PLGA-SS-mPEG. Results infer that the interaction of SQ890 NPs with Fe^3+^ ions diminished its absorption, indicating the successful chelation of Fe ions, which could then be recovered upon exposure to GSH, which reduces Fe^3+^ to Fe^2+^ ions (Supplementary Figure S2). The morphological properties of SQ890@Fe NPs were then detected by transmission electron microscopy (TEM) and dynamic light scattering (DLS). SQ890@Fe NPs displayed uniform spherical shapes of ∼134 nm with good dispersion and kept a stable particle size during a 7-days storage period, and the zeta potential of SQ890@Fe NPs was determined to be −10.08 mV (Figures 2C–E), indicating favorable morphological features to aggregate at tumor sites through the EPR effect. For PTAs, the photothermal property of SQ890@Fe NPs is pivotal for the application of PTT. To further evaluated the photothermal properties of SQ890@Fe NPs, temperature changes and infrared photothermal images were recorded under 808 nm laser illumination (1 W cm^−2^), with or without adding GSH. The results indicate that SQ890@Fe NPs showed a negligible temperature increase in 300 s under irradiation. As expected, upon treatment with GSH, a significant rise occurred in the absorbance, and the temperature of SQ890@Fe NPs increased significantly upon 808 nm light irradiation. The temperature increments positively correlate with laser power density and concentration of SQ890@Fe NPs (Figures 2F,G). The infrared thermal images recorded using a thermal cameral are shown in Figure 2H. Negligible temperature changes after five “laser on–off” cycles validate the excellent photothermal stability of SQ890@Fe NPs (Figures 2H,I). Furthermore, according to heating and cooling curves and the linear relationship between cooling time and -ln(θ), the photothermal conversion efficiency of SQ890@Fe NPs was calculated to be about 54% (Figures 2J,K). Besides the photothermal properties, ferrous ions released from SQ890@Fe NPs was measured in the presence of GSH (Figure 2L). The results showed that upon treatment with GSH (10 mM), Fe ions released from SQ890@Fe NPs is gradually and tremendously increased with time. The disulfide bonds in PLGA-SS-mPEG cleaved in the presence of GSH, and the SQ890-Fe^3+^ complexes were released. Meanwhile, Fe^3+^ ions were reduced to Fe^3+^ ions by GSH. Both the two processes would consume the GSH in cancer cells, which could attenuate the ROS scavenging and enhance the cancer cell killing effect. Moreover, the GSH-activated nanomedicine offers an accurate and efficient antitumor effect at tumor sites, avoiding potential tissue damage.

### Measurement of cellular uptake *in vitro*

**FIGURE 3:**
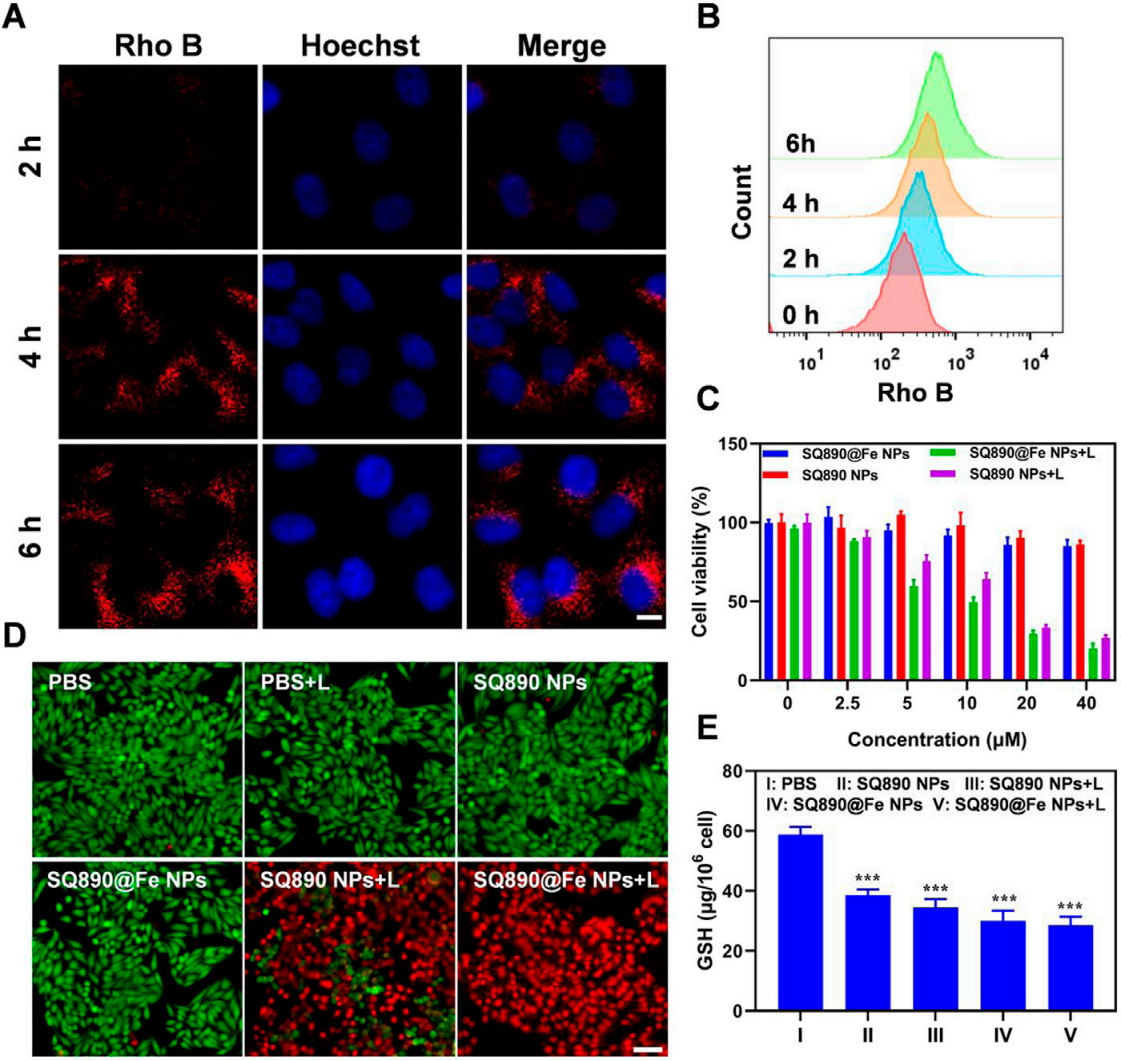
Cellular uptake and cytotoxicity to HepG2 cells. **(A,B)** Cellular internalization of nanoparticles labeled with Rho B observed by CSLM **(A)** and quantitative analysis by flow cytometry **(B)**. Scale bar: 10 μm. **(C)** Viability of HepG2 cells after treated with different concentrations of SQ890@Fe NPs or SQ890 NPs in dark or under 808 nm laser illumination. **(D)** Live/dead stained by PI and calcein AM of HepG2 cells after various treatments. **(E)** The GSH in HepG2 cells after various treatments (***p < 0.001 versus PBS, *n* = 3/group).

Rho B NPs were prepared using the above methods to measure the cellular uptake of NPs. After HepG2 cells were treated with Rho B-labeled NPs for 2, 4, and 6 h, the cellular uptake was measured by flow cytometry and visually determined using a confocal laser scanning microscopy (CLSM). The results show a time-dependent gradual increase in the Rho B-labeled NPs internalized by HepG2 cells (Figure 3A). As shown in Figure 3A, Rho B-labeled NPs were significantly internalized by HepG2 cells at 4 h and emitted strong red fluorescence, which increased at 6 h, indicating the highest level of NPs internalization. These observations were further validated by the flow cytometric analysis (Figure 3B). These results showed that Rho B-labeled NPs were readily taken up by HepG2 cells, and that this uptake increased over time, demonstrating Rho B-labeled NPs’ potential to infiltrate cancer cells and guiding the timing of irradiation.

### *In vitro* cytotoxicity assay

Motivated by the photothermal properties of SQ890@Fe NPs, standard MTT assays were conducted to measure the cytotoxicity and biocompatibility. According to the MTT results, SQ890@Fe NPs and SQ890 NPs showed slight cytotoxicity at a relatively high concentration (40 μM) in dark. However, when exposed to 808 nm lasers, the cell viability of HepG2 cells treated with SQ890 NPs and SQ890@Fe NPs decreased rapidly with the increased NPs concentration (Figure 3C). Live/dead cell staining was conducted to intuitively observe the cell viability by an inverted fluorescence microscope (Figure 3D). Cells treated with both laser illumination and NPs emitted strong red fluorescence indicating dead cells, while nearly all cells in monotherapy groups stained green representing alive cells, thus signifying the biosafety and high PTT efficacy of the NPs. Furthermore, the GSH level was measured in cells. As demonstrated in Figure 3E, GSH levels declined in all the groups treated with NPs in monotherapy and combination therapy with laser irradiation. The SQ890@Fe NPs showed a noticeable decrease relative to SQ890 NPs, which further decreased upon laser irradiation. These findings indicate the efficacy of SQ890@Fe NPs in high level GSH environment, where GSH is utilized to reduce Fe^3+^ to Fe^2+^ to induce ferroptosis. Likewise, the treatment of HepG-2 cells with different concentrations of SQ890@Fe NPs showed a gradual concentration-dependent rise in the LPO production (Supplementary Figure S3), which resulted in ferroptosis.

### *In vivo* therapy of tumor

**FIGURE 4:**
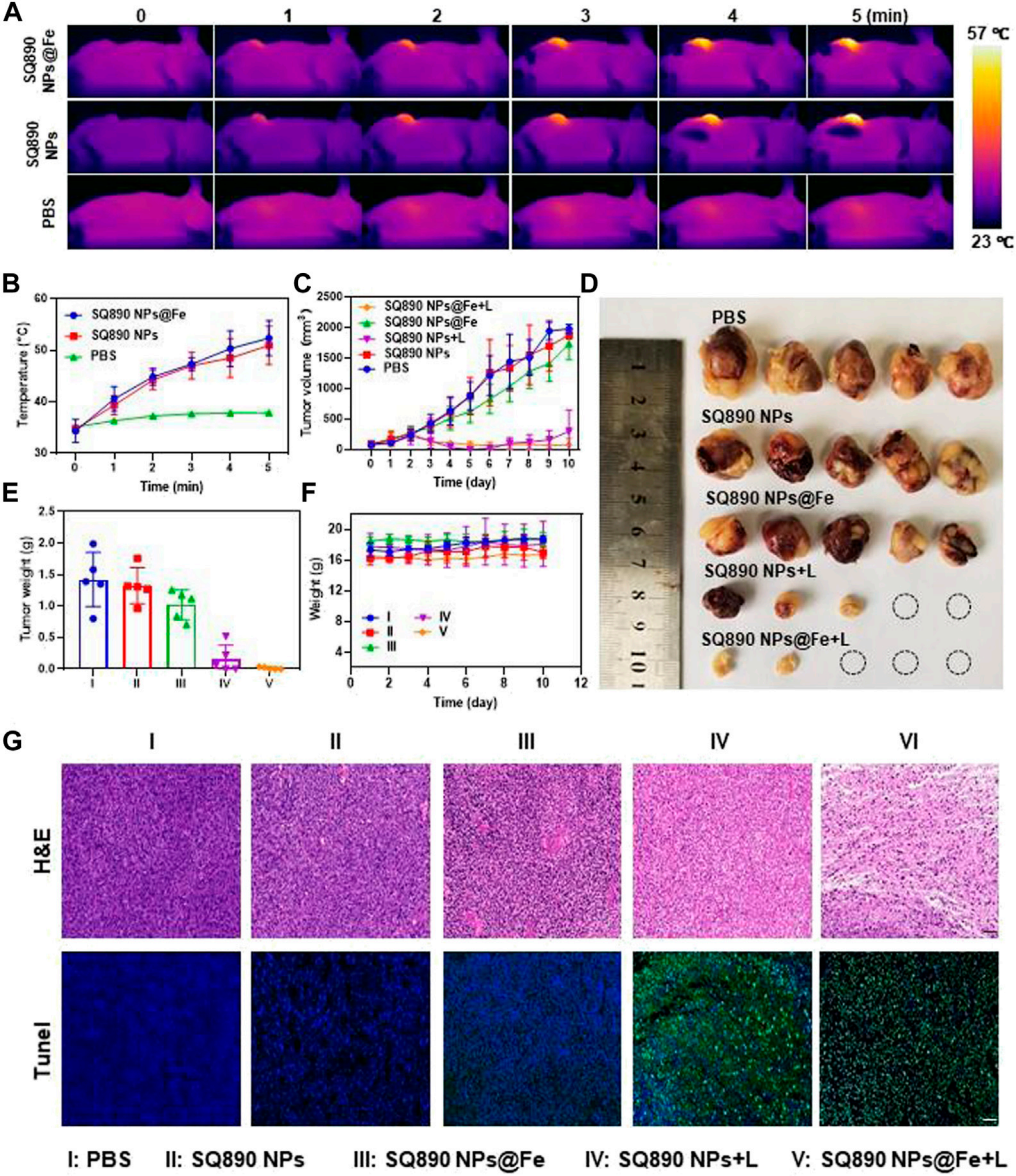
*In Vivo* antitumor effects. **(A,B)** IR thermographic pictures **(A)** and temperature changes of mice **(B)** administrated with PBS, SQ890@Fe NPs, and SQ890 NPs exposed to 808 nm laser (1 W cm^−2^). **(C)** Tumor volume growth curves of mice during the 10-days treatments (*p < 0.05, ***p < 0.001 versus PBS, *n* = 5). **(D)** Photos of tumors after excision at the termination of treatment. **(E,F)** The tumor **(E)** and body **(F)** weights of mice in each group (***p < 0.001 versus PBS, *n* = 5). **(G)** H&E and TUNEL staining of tumors after 10-days treatment. Scale bar: 20 µm.

Inspired by the above results, we further explored the tumoricidal efficacy of SQ890@Fe NPs *in vivo* in mice with subcutaneous PDX^HCC^. At 12 h postinjection, mice in laser therapy groups were anesthetized and then illuminated with 808 nm light for 5 min and the fluctuations in tumor temperature were recorded. The tumor site temperatures in the SQ890 NPs + L group and SQ890@Fe NPs + L group reached 50.9°C and 52.3°C in 5 min, respectively, which led to tumor ablation. However, the temperatures were mildly raised to 37.9°C in mice treated with PBS (Figures 4A,B). The tumor volume was then recorded for 10 days to assess the tumor growth (Figure 4C). Results show that tumor growth in SQ890@Fe NPs treated group was slowed down relative to SQ890 NPs and PBS. Moreover, the combined treatment groups with laser irradiation, not only prevented the tumor growth tremendously, but also regressed the tumors in SQ890 NPs + L (2 mice) and SQ890@Fe NPs + L (3 mice) treated groups. Further, excised tumors were imaged and tumor weights were recorded, which further validated the efficacy of the treatment (Figures 4D,E). Thereafter, the biological safety of the SQ890@Fe NPs was measured. Negligible changes occurred in the body weight of mice across treatment groups (Figure 4F). Furthermore, the hematoxylin and eosin (H&E) and terminal deoxynucleotidyl transferase uridine triphosphate nick end labeling (TUNEL) staining of tumor tissues revealed obvious damage (apoptosis) in tumor cells of mice from laser treated groups (Figure 4G), suggesting a distinct therapeutic effect.

Safety is an essential aspect to consider in the development of effective therapeutic drugs. The serum analysis of biochemical parameters (CK-MB, UA, CREA, ALT, AST) was performed to assess the cytotoxicity of nanoparticles treatment. As shown in Supplementary Figure S4 **,** the level of ALT and AST, was unchanged in the SQS890@Fe NPs treated group compared to vehicle treated group, suggesting the safety of nanoparticles on liver function. In addition, the level of creatinine and UA remained within the normal ranges, implying that SQS890@Fe NPs treatment has no toxicity to kidney. Consistently, SQ890@Fe NPs treatment does not cause a marked increase in the serum LDH and Ck-MB levels. In line with these results, the standard H&E coloration showed that no pathological changes were found in all major organs after the treatment period (Supplementary Figure S5). Collectively, the SQ890@Fe NPs orchestrated ferrotherapy and PTT synergistically, which displayed significant antitumor effects*.* The demonstrated antitumor efficacy in our study, and lack of toxicity bodes well for the further clinical development of SQ890@Fe NPs as a potential antitumor drug.

## Conclusion

In summary, we synthesized a novel GSH activatable, Squaraine-based nanomedicine that is used for PTT and ferrotherapy. Chelation of Fe^3+^ by the SQ890, significantly reduced the absorption of SQ890, which was tremendously increased upon GSH treatment, allowing for PTT. By assembling into NPs with GSH sensitive polymer PLGA-SS-mPEG, the SQ890@Fe NPs showed GSH-activated PTT and ferrotherapy for HCC. In the TME, the photothermal effect was activated by the high level of GSH, which could enhance the formation of OH radicals and LPO due to the increased Fenton reaction rate. Likewise, the heat-induced HSPs were further limited by the increased radical, which preventing the self-protection mechanism in tumor. Moreover, the GSH-activated release of Fe^2+^ and photothermal effect in the TME may provide an intelligent paradigm for HCC therapy. As a result, the GSH-activatable medicine SQ890@Fe NPs combined PTT and ferrotherapy and exhibited a significant anti-tumor effect both *in vivo* and *in vitro*. Taken as a whole, SQ890@Fe NPs holds the promising potential to be an effective novel therapeutic agent in combined therapy, providing a forward step in designing a combination regimen for cancer therapy.

## Data Availability

The original contributions presented in the study are included in the article/Supplementary Material, further inquiries can be directed to the corresponding authors.
