# Supplementary material for: A smart nanoplatform for enhanced photo-ferrotherapy of hepatocellular carcinoma
Source: Front Bioeng Biotechnol. 2022 Sep 20;10:1022330. doi: 10.3389/fbioe.2022.1022330 (PMC9530464; doi:10.3389/fbioe.2022.1022330)
Supplement: Supplementary file 1 [file DataSheet1.pdf]

Supporting Information

A Smart Nanoplatform for Enhanced Photo-ferrotherapy of Hepatocellular Carcinoma

Longguang Tang[1,2#*], Mingjian Ling[3#], Madiha Zahra Syeda[4#], Rui Sun[5], Minghui He[6], Qingchun Mu[1], Xiulong Zhu[1], Chunming Huang[1*], Liao Cui[2*]

[1]The People's Hospital of Gaozhou, Guangdong Medical University, Maoming 525200, China
[2]Guangdong Provincial Key Laboratory of Research and Development of Natural Drugs, and School of Pharmacy, Guangdong Medical University, Dongguan 523808, China
[3]Southern Medical University Shenzhen Stomatology Hospital (Pingshan), Shenzhen 518000, China
[4]Department of Pharmacology and Department of Respiratory and Critical Care Medicine of the Second Affiliated Hospital, Zhejiang University School of Medicine, Key Laboratory of Respiratory Disease of Zhejiang Province, Hangzhou 310009, China
[5]School of Pharmaceutical Sciences, Guangdong Provincial Key Laboratory of New Drug Screening, Southern Medical University, Guangzhou, China
[6]Key Laboratory of Growth Regulation and Translational Research of Zhejiang Province, School of Life Sciences, Westlake University, Hangzhou, Zhejiang, China

[*]Corresponding author:
Email: tanglongguang@zju.edu.cn (L.G. Tang); Huangchunming5888@163.com (C.M. Huang); cuiliao@gdmu.edu.cn (L. Cui)
[#]Authors contributed equally.

Experimental Methods

Characterization

Synthesis of SQ890-Fe^{2+} and SQ890-Fe^{3+}

We first synthesized the SQ890 molecules as described previously. Briefly, SQ8090 was fabricated through a three-step reaction, namely electrophilic substitution, Grignard Reaction, and condensation reaction. Then SQ890-Fe^{3+} and SQ890-Fe^{2+} was prepared by mixed SQ890 (1mmol) with $FeCl_3$ (2 mmol) and $FeCl_2$ (2 mmol) for 2 minutes, respectively, in THF solution.

Preparation of SQ890@Fe NPs

The SQ890@Fe NPs was prepared through a nanoprecipitation method by the self-assembling of SQ890-Fe^{3+} and the GSH sensitive polymer PLGA-SS-mPEG. SQ890-Fe (3 mg) and PLGA-SS-mPEG (100 mg) was fully dissolved in CH_2Cl_2. After the solvent was removed by thin-film dispersion method, ultrapure water (3 mL) was slowly added and ultrasonic dispersion was performed. SQ890@Fe NPs was obtained after dialysis purification and stored at 4 ℃ for further use. All chemicals were purchased from Alfa Aesar, Thermo Fischer Scientific, or Sigma-Aldrich and used without further purification. Ultrapure water was prepared using a Milli-Q Plus System.

Photothermal measurement

Various concentrations of SQ890@Fe NPs solution (1 mL), with GSH (10 mM), were illuminated with 808 nm light at various power densities to evaluate the photothermal properties, and the corresponding thermographic images were recorded using an IR camera.

For the photothermal conversion efficiency (η) evaluating, 1 mL SQ890@Fe NPs and 1 mL water were illuminated with 808 light for 500 s until reaching the maximum stable temperature and then spontaneous cooling to room temperature. The temperature variations were recorded and calculated.

For photostability tests, 1 mL SQ890@Fe NPs was irradiated for 5 min and cooled for 5 min, repeated these cycles 5 times, and recorded the temperature variation.

Reagents and Cell line and Measurement of cellular uptake *in vitro*

Rho B was assembled into NPs following the above mentioned methods. HepG2 cells (2.5 × 10^5) were seeded in 6-well plates for 12 h. After being cultivated with DMEM medium containing Rho B NPs (10 μM) for 2 h, 4 h, and 6h, the cells were washed with PBS and fixed with 4 % (W/V) paraformaldehyde . Then the cells were washed PBS and stained with Hoechest 33342 (2 μg/mL, blue) for 5 min. And the cellular uptake was visually determined using CLSM.

Cell viability assay *in vitro*

HepG2 cells (5 × 10^3) were seeded in 96-well plates and cultured 12 h for attachment. Thereafter, cells were treated with various concentrations of SQ890 NPs and SQ890@Fe NPs and illuminated with/without 808 nm light (1 W/cm^2) for 5 min at 4 h post-administration. The cells were then maintained for another 20 h. The cell viability was measured by MTT assay. All cell lines were purchased from American Type Lifestyle Collection (ATCC).

***In Vitro* Fluorescent imaging**

HepG2 cells were incubated in a DMEM medium containing SQ890 NPs (20.0 μM) and SQ890@Fe NPs (20.0 μM) for 4h, the cells were then illuminatied with/without 808 nm light (1 W/cm^2) for 5 min. Thereafter, Calcein AM/PI buffer was added after washing the cells and cultivated for 30 min in dark. Then using an inverted fluorescent microscope to record the cells fluorescent images immediately.

Measurement of intracellular LPO level

Intracellular Lipid Peroxidation. HepG-2 cells were seeded into 6-well plate (1×106 cells/ well) and treated with PBS, SQ890@Fe NPs (25 μM), and SQ890@Fe NPs (60 μM) cultivated for 24 h. Next, after digested, resuspended, and fragmented, they were then centrifuged at 10000 rpm for 10 min and the supernatants collected. A commercial assay kit (Lipid peroxidation MDA assay kit) was used to evaluate intracellular LPO levels by UV-vis spectroscopy.

Animal models. The animal experiments were performed following a protocol approved by the Animal Care and Use Committee of Guangdong Medical University. The 5-6 weeks female

BALB/c nude mice and Kunming mice were supplied by Guangdong Sijiajingda Biotechnology Co., Ltd. 5-6-week-old female BALB/c nude mice were used to establish the PDX^{HCC}. Blocks of tumor tissues (about 3×3×3 mm^3/ block) were subcutaneously inoculated in the right hind leg of BALB/c nude mice.

***In vivo* PTT**

The tumor-bearing mice were randomly divided into 5 groups (n=5). When the tumor volume reached about 80-100 mm^3, mice were administrated with PBS, SQ890 NPs (5 mg/kg) and SQ890@Fe NPs (5 mg/kg) and illuminatied with/without 808nm light (1 W/cm^2) for 5 min at 8 h post-administration. During irradiating, thermographic pictures of mice were collected using an IR camera. Recording the tumor size and bodyweight every two days during the 10-day treatment period. At the termination of treatment, mice from each group were sacrificed to gathered the tumors and major organs for TUNEL and H&E staining, and blood samples for the hematology analysis.

Blood biochemistry analysis

Blood was collected from the heart of tumor bearing mouse models in different treatment groups, and centrifuged for 15 min at 1,500g, 4 °C. Serum was isolated and collected in the fresh tubes. BUN, CREA, ALT, and AST in the serum were quantified to indicate the renal and hepatic function

Statistical analysis

Experimental data were analyzed using GraphPad Prism 9 or SPSS software. All data are expressed as the mean ± SEM. Statistical differences between groups were analyzed using independent two-sample t-tests. Differences between groups were considered significant at $P < 0.05$.

Supplementary figures:

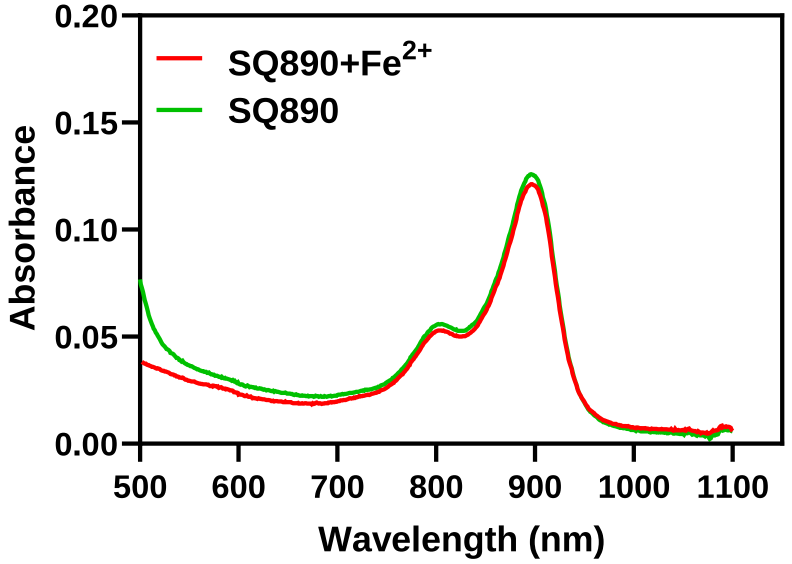

Figure S1. The absorbance of SQ890 incubated with or without Fe^{2+}.

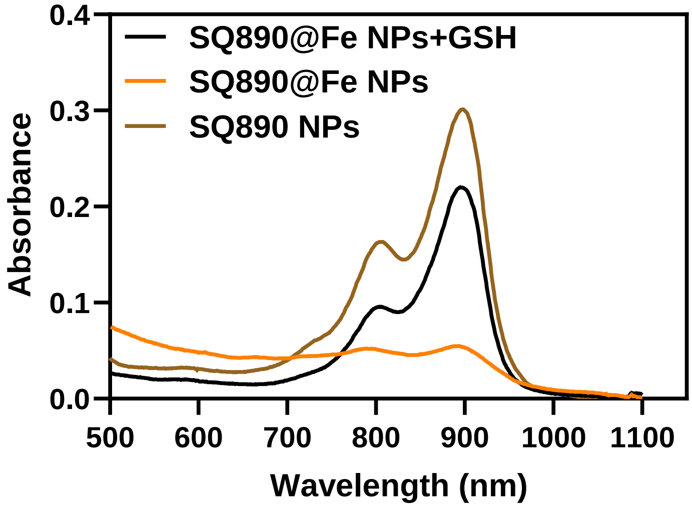

Figure S2. The absorbance of SQ890 NPs and SQ890@Fe NPs treated with or without GSH.

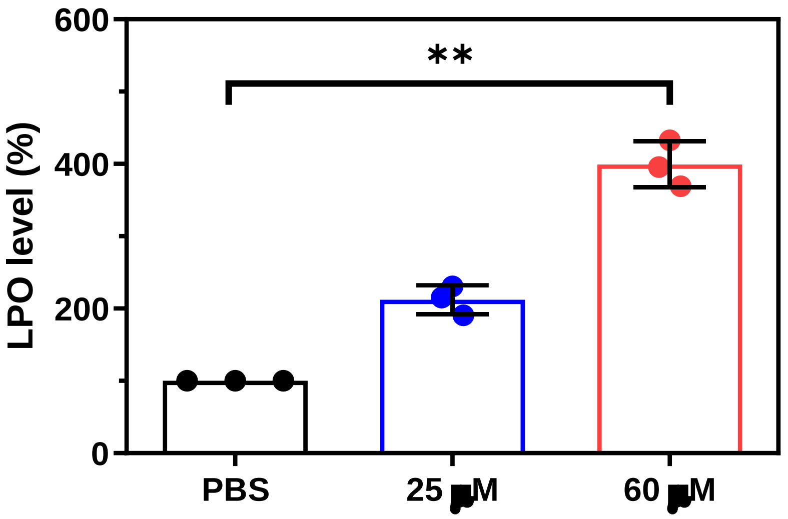

Figure S3. Intracellular lipid peroxidation in HepG-2 cells treated with SQ890@Fe NPs at different concentrations

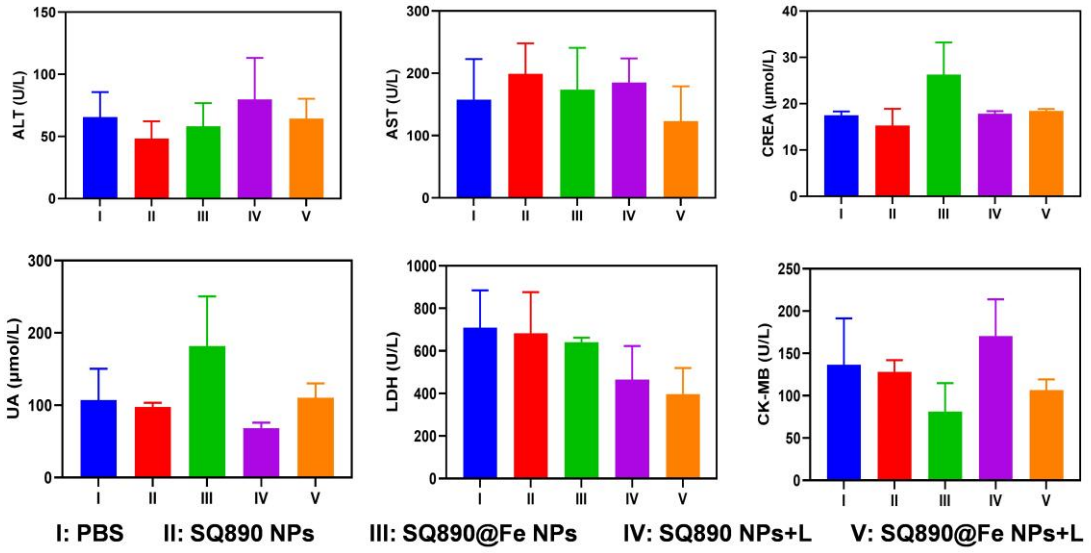

Figure S4. Blood biochemistry analysis of mice. The examined parameters included CK-MB, AST, ALT, UA and CREA.

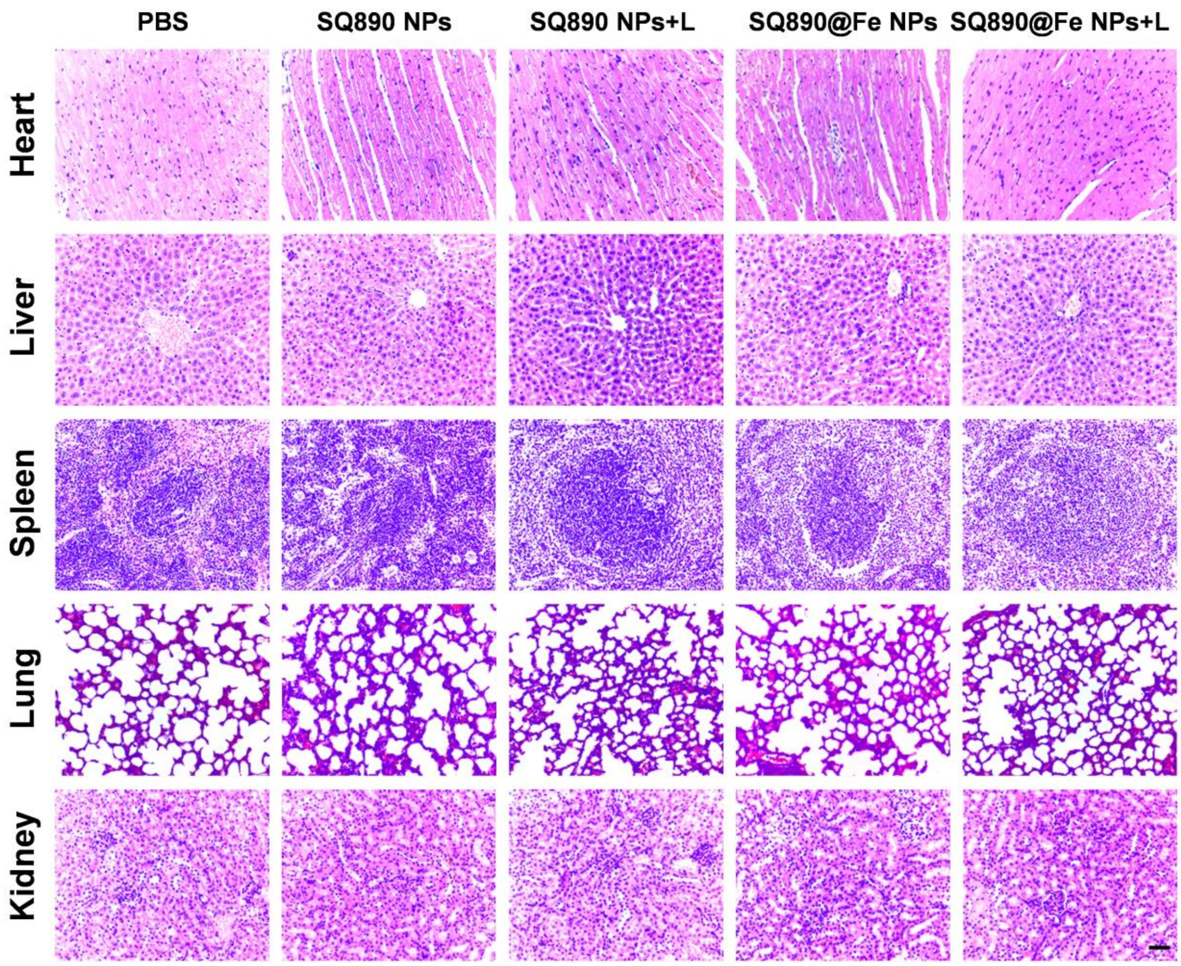

Figure S5. H&E staining of mice organs. H&E-stained slices obtained from the heart, liver, spleen, lung, and kidney of mice after various treatments. Scale bar: 25 μm.
